# Impacts of cigarette smoking on blood circulation: do we need a new approach to blood donor selection?

**DOI:** 10.1186/s41043-023-00405-2

**Published:** 2023-07-05

**Authors:** Jie Wang, Yuhan Wang, Weixin Zhou, Yuanshuai Huang, Jianbo Yang

**Affiliations:** 1 Department of Transfusion, The Affiliated Hospital of Southwest Medical University, Luzhou, 646000 Sichuan Province People’s Republic of China; 2 Department of Laboratory Medicine, Luzhou Longmatan District People’s Hospital, Luzhou, 625000 Sichuan Province People’s Republic of China; 3 Department of Nuclear Medicine, The Affiliated Hospital of Southwest Medical University, Luzhou, 646000 Sichuan Province People’s Republic of China

## Abstract

Smoking is a major public health problem and is considered the leading cause of preventable death worldwide. Gas-phase smoke carries bioactive substances and toxic compounds, affecting human health and reducing life spans. The negative effects of smoking on red blood cell (RBC) quality include destroying RBCs and increasing carboxy hemoglobin (COHb). Smoking increases the concentrations of heavy metals such as cadmium (Cd) and lead (Pb) in the blood. Moreover, tobacco smoking has been found to be associated with heightened platelet (PLT)-dependent thrombin level which will induce a prothrombotic state. Smoking may affect the blood circulation of donors, and subsequently the blood components, and ultimately the recipients of transfusion. Nevertheless, there are no restrictions on smoking for volunteer blood donor screenings currently. We reviewed the articles about the influence of smoking on smokers' blood circulation as well as the impact of donated blood products on transfusion when these smokers act as blood donors. We aim to attract blood collection centers’ attention to strengthen the management of blood donors who smoke, avoiding their use in massive transfusion protocol and susceptible recipients, especially pediatric ones.

## Introduction

Currently, the number of deaths because of smoking is 7 million each year, of which 6 million are active smokers while around 0.9 million are passive smokers [1]. The death toll is expected to rise to 10 million by 2030 [2]. Gas-phase smoke carries bioactive substances such as carbon monoxide (CO), ammonia, ketones, formaldehyde, acetaldehyde, and acrolein, whereas particulate matter carries nicotine; heavy metals such as nickel, arsenic, cadmium (Cd), and lead (Pb); and other constituents such as benzopyrenes [3]. Most of these products are toxic, causing tissue damage secondary to oxidative stress and inflammation [4]. Given that humans are oronasal inhalers, the inhaled smoke travels through the respiratory as well as the digestive tract to enter blood circulation, affecting plasma, blood cells and tissues; causing oxidative damage [5]; affecting human health; and reducing life spans.

Although public efforts to discourage smoking have been successful in reducing smoking rates, consumption of other forms of nicotine is on the rise, in the form of e-cigarettes, an alternative that claims to be healthier and safer than traditional tobacco [6–8]. E-cigarettes, also known as vape pens, e-cigars or vaping devices, are electronic nicotine delivery systems that produce an aerosol mixture of flavored liquid and nicotine for the user to inhale [9]. It was reported that about one-quarter of U.S. youth and young adults have tried e-cigarettes [10, 11]. The main toxic constituents in e-cigarette liquids or vapors are carbonyl compounds, nicotine, and particulate matter [12]. According to one study, e-cigarettes emit chemicals at levels comparable to tobacco smoke [13]. Despite the full implementation of comprehensive tobacco prevention and control strategies, coupled with the U.S. Food and Drug Administration’s (FDA) regulation of tobacco products, the number of young users, especially high school students, of tobacco products remains high [14, 15]. They represent an important blood donor demographic [16].

Blood transfusion is the oldest and most common therapeutic modality for hemorrhage and anemia. As with other therapies, transfusion is associated with the risks of adverse effects, morbidity, and mortality for the recipients [17]. Serious adverse events such as multiple organ failure, renal dysfunction, and death can also occur [18]. Ensuring the safety of the blood supply is important in transfusion. To safeguard the quality of blood products, the FDA and the Association for the Advancement of Blood and Blood Products (AABB) have established requirements to determine whether the individuals are suitable donors [19, 20]. These requirements are designed to protect recipients and donors by excluding the donors who might pose risks to blood transfusion [16]. For example, doctors and clinics should identify and exclude people who are exposed to infectious diseases or drugs such as anticoagulants, chemotherapy, or potential teratogens [16] via a health history interview and a physical examination, as well as hematological and serological testing [21].

On July 27, 2021, the World Health Organization (WHO) launched its eighth report on the global tobacco epidemic. It noted that worldwide smoking prevalence among people older than 15 years stood at 17·5% in 2019 [22]. Despite the prevalence of tobacco use worldwide, smoking habits are not carefully investigated prior to blood donation and little literature was found reporting the impact of smoking on blood transfusion. The prevalence of smoking among blood donors and the effect of cigarette smoke exposure on the quality of donated blood lack in-depth exploration. The concerns regarding the lack of abstinence period for smokers in the existing blood donation guidelines before donating blood merit justified attention [23]. The aim of this review is to summarize the literature about the influence of smoking on smokers' blood circulation as well as the impact of donated blood products on transfusion when these smokers act as blood donors.

## Smoking destroys red blood cells and increases nicotine and carboxy hemoglobin levels

The main effect of smoking on the RBCs of smokers is the destruction of membranes [24–26]. Particles of micron and nanometer size, heavy metals, radioactive substances, free radicals, and peroxides present in inhaled cigarette smoke damage cell membrane surfaces by pitting and etching [26]. The targets are proteins such as collagen, elastin, and the lipids found plentifully on the surfaces of red cells. This surface damage allows toxins such as benzene, nicotine, CO, and hydrogen peroxide to enter the RBCs [27]. Chronic cigarette smoking (more than one pack per day and COHb > 3%) alters RBC membrane deformability [28]. The fatty acid analysis of the individual phospholipids showed a preponderance towards saturated fatty acids in smokers who smoked tobacco in any form, equivalent to 10–40 cigarettes per day or a total of 2–15 pack years, suggesting tighter membranes, which finally affect the signal transduction mechanism and cell response [29]. Consequently, the RBC membranes become deformed, with pointed extensions, swellings, fissures, crater-like structures, and losses of the discoid shape [24]. In addition, the exposure to toxins and peroxidants causes hemolysis as well as loss of membrane stability [30]. As a result, the RBCs of smokers are more fragile, which contributes to the decrease in RBC count and Hb [31], causing numerous diseases associated with anemia in smokers [32]. Meanwhile, the O_2_-carrying capacity of the RBCs is reduced [1]. Transfusion of RBCs from smokers whose average consumption were at least 20 cigarettes per day will lead to impaired oxygen delivery, detracting from the main purpose of the blood transfusion [33].

The sustained production of reactive free radicals from the tar and gas phases of cigarette smoke causes oxidant stress on circulating RBCs [21, 34]. Mature RBCs are unable to resynthesize antioxidants due to their continuous exposure to xenobiotics from cigarette smoking. As a consequence, RBCs from smokers may have a higher risk of storage lesion than those from non-smokers [33]. Moreover, studies have revealed that 50% of the RBCs from smokers had lower uric acid levels, making these bags more susceptible to storage lesions [35]. However, whether the RBC lesions affect the safety and effectiveness of blood transfusions remains controversial [25, 36–38].

Nicotine is a toxic and addictive substance in tobacco. Cotinine is the main metabolite of nicotine, which has a much longer half-life in vivo than nicotine (about 16 h for cotinine and 1–2 h for nicotine [39]). In a pilot study, patients who received cotinine-positive RBC units showed a significantly reduced hemoglobin (Hb) increment (median + 0.4 g/dL, IQR 0–1.3 g/dL) compared with those who received cotinine-negative RBC units (median + 1.4 g/dL, IQR 1.1–1.9 g/dL) (*p* = 0.014) [40]. Furthermore, for red blood cells donated by smokers are more vulnerable to radiation damage, recipients of gamma-irradiated RBC units from smokers were more likely to require additional transfusions compared to recipients of RBC units from nonsmokers [41].

Cigarette smoking is a major source of CO in blood products, and COHb levels are significantly higher in RBC units donated by smokers than in those donated by non-smokers [42]. A study found that among 410 blood donors, about 6% had increased CO levels due to smoking [16, 43]. Another study has shown that smoking among donors leads to higher levels of COHb in blood circulation as well as in blood products [44]. Given that the hematocrit (Hct) in the bags is higher than in donor’s blood in non-additive solution, COHb level is higher in smokers’ donated units [43]. As CO has more than 210 times the affinity of Hb with oxygen (O_2_) [21], carboxy hemoglobin (COHb) is produced in large amount, causing the hematoporphyrin in Hb to break up and rush into the plasma stream. Ultimately, the normal lifespan of RBCs is reduced from 120 days to 80–85 days due to smoking [26]. Meanwhile, Hb affinity to CO reduces the O_2_-carrying capacity of tissues, leaving less O_2_ available for delivery to the tissues, causing hypoxia and acidosis [45].

Transfusion of RBC containing higher COHb content may be insignificant for most adults as COHb will be diluted in the total blood circulation [21, 40]. However, a recipient who requires larger volumes of blood, as through massive transfusion protocol (MTP), during major surgery, or in newborns may be adversely affected [46–50]. A case reported COHb levels reached 3.7% from a newborn due to the transfusion of a single RBC pack from a smoker containing 7.2% COHb during a cardiopulmonary bypass circuit. As a result, the patient developed acute hypoxia. Luckily, the potential risk of impaired O_2_ delivery was successfully reversed by administration of 100 percent inspired O_2_ [45, 46]. In view of this, the research team concluded that only those RBCs with COHb levels of < 1.5% are acceptable for pediatric cardiac surgery [45].

In short, donor smoking has detrimental consequences on RBC units, which will influence the safety and efficacy of transfusion.

## Smoking increase cadmium and lead concentrations in blood

Smoking is considered a source of toxic elements [51, 52]. Cd and Pb concentrations are significantly higher in blood from smokers compared to non-smokers. Cd is not detected in blood from non-smokers, but it reaches the value between 1 and 2 μg/L in blood from smokers [53]. Cd, which is considered a human carcinogen (Group 1) [54], enters the bloodstream through absorption from environmental sources and is stored in tissues, where it remains for up to 10–35 years [54, 55]. Pb is classified as possibly carcinogenic (Group 2B) in humans by the International Agency for Research on Cancer (IARC) [56]. In adults, almost 100% of Pb is excreted from the body within a few weeks once it is absorbed. During the same period, however, only about 30% of Pb leaves a child's body, accumulating instead in the bones, where it remains for decades [57]. In children, Pb is associated with impairments to physical development, learning, and memory [58, 59]. Even when blood concentrations are below 100 μg/l, Pb can also cause cognitive, behavioral, and psychological disorders and induce irreversible neurological impairment [60]. In 2012, the US Centers for Disease Control proposed that there were no safe levels of Pb for children [61].

Many of the toxic substances presented in cigarettes transfer into the blood, subsequently to blood components, and ultimately to transfusion recipients [62, 63]. In all non-smokers’ blood, Pb levels are below the limit (ranging from 4 to 43 µg/l), whereas 5 of the 36 blood samples from smokers have Pb levels higher than 50 µg/l (ranging from 51 to 72 µg/l) [53]. Transfusion of blood components has been recognized as a major source of Pb exposure in pediatric recipients [64, 65]. Given that children's detoxification mechanisms are still immature, it is recommended that they avoid unnecessary exposure to toxic elements, such as blood transfusions from smokers. RBCs from one donor are divided into pediatric packs, and the same child can receive RBCs from a single donor in different transfusions [66]. Therefore, by having multiple transfusions from the same smoker, the pediatric patient can receive potentially toxic elements [53].

In sum, toxic elements levels are higher in blood components from smokers. Avoiding the use of smokers’ blood on children contribute to the improvement of pediatric blood transfusion safety.

## Impacts of smoking on platelet dependent thrombogenesis

Literature that covers the effects of smoking on PLT components and transfused recipients are countable so far. Besides, the effects of smoking on PLT function are controversial. Studies have indicated that smoking enhanced PLT activity, but the most significant influence existed only briefly [67, 68]. Chronic smoking (more than 10 cigarettes per day for at least 7 years) results in increased PLT count [69, 70], PLT adhesiveness, and aggregation [71–74]. Smokers are prone to develop hyperlipidemia and hypercholesterolemia [75], which causes an increased cholesterol phospholipid ratio (C/P) of membrane and decreased PLT membrane fluidity [71]. The PLT from a smoker showed changes to the smoothness of the membrane by electron microscopy, such as bulbous swellings and open canalicular pores, contributing to reduced fluidity of the PLT membrane [76]. Consequently, the PLT aggregation and secretion were enhanced [71, 77].

PLT activation and enhanced PLT aggregation cause thrombin stimulation and fibrin formation [71, 78, 79]. In 2001, Hioki et al. [80] found the PLT-dependent thrombin level was significantly higher in healthy male smokers than in non-smokers at baseline, although the smokers had abstained from smoking for at least 12 h. Besides, chronic smokers had a heightened PLT binding to fibrinogen and PLT-dependent thrombin generation levels even after abstaining from smoking for 4–6 h [81, 82]. In an ex vivo observation, decreased fiber thickness and increased fibrin fiber density from postsmoking samples were seen as against to presmoking and non-smoking samples using an electron microscope [83].

As cigarette smoke gas phase contain toxicants [3], internal environment is affected by these toxins, disrupting homeostasis. Oxidative stress induced by reactive oxygen species (ROS) and inflammation-derived oxidants modulates PLT function and change the coagulation system [71]. Furthermore, increased nicotine and cotinine levels caused by smoking induce a prothrombotic state in smokers by increasing PLT-dependent thrombogenesis [80], which could progress to coronary artery thrombosis.

To sum up, smoking produces a prothrombotic phenotype, that is, enhanced PLT aggregability and subsequent alterations in the clotting cascade, which are pathophysiological events of cardiovascular diseases in smokers themselves [78, 79].

Although smoking changes hemostatic systems, its detrimental effects on packed plateletpheresis products and recipients are indefinite [84, 85]. Therefore, there is no need to exclude smokers from PLT donations [84]. The clinical impact of smoking on platelet transfusion warrants further research.

## The management of smokers in blood donation

The advent of “vein to vein” databases linking information from blood collection facilities and transfusion services to patient outcomes is a growing subject of study [86]. Health concerns in blood donors may affect the quality of donated blood products [87]. According to the WHO, safe and adequate blood should be an integral part of every country’s national health care policy [88]. Despite efforts to improve safety, transfusion is associated with risks of adverse events, morbidity, and mortality for recipients [17, 88]. The impact of cigarette exposure on the quality of donated blood components, as well as transfusions’ efficacy in recipients, have not been extensively explored. The data on effects of smoking in blood components and storage lesions are ambiguous.

The time interval between smoking and blood donation seems to be a particularly important factor related to the CO concentration in the blood [43]. Some authors indicated that cigarette abstinence for at least 24 h before blood donation could have a positive effect [89]. In 2018, Boehm et al. [21] found that abstaining from smoking for 12 h was enough to reduce COHb levels and consuming less than 20 cigarettes a day was associated with lower cotinine levels. Unfortunately, implementing smoking restrictions as prerequisites for blood donation seems impracticable, as such advocacy may decrease blood donation from tobacco-dependent individuals.

However, smokers are less likely to donate because smoking could potentially predict comorbidity and loss of eligibility [90]. Blood donation is associated with better health outcomes in donors because of the Healthy Donor Effect (HDE), referring to the fact that donors are a selected “healthier” subset of the general population, subject to both donor selection procedures and self-selection [91–93]. A study in the Netherlands reported that active whole blood and plasma donors were significantly less likely to be current smokers than the general population (17.1% of blood donors were current smokers as opposed to 27.3% of the general population, odds ratio 0.55, 95% confidence interval 0.49–0.62) [94]. A study found that 5.9% of whole blood donors in the Hospital de Clinicas de Porto Alegre Blood Bank were smokers, which was a lower percentage than in the general population of Porto Alegre (14.2%) [21]. Another retrospective cohort study reported that the rate of smoking in RBC donors was 6.4% in California, lower than the smoking rate in the United States (15.5%) [41]. An investigative study from Vanderbilt University Medical Center found that 19% of tested RBC units contained detectable concentrations of nicotine or its metabolites, consistent with national trends in tobacco use (15.5%) [16]. Another investigation in Norway reported a lower prevalence of smoking in PLT donors (5% of donors were smokers as opposed to 13% of the general population) [95].

In summary, smoking is not routinely captured in donor questionnaires before donation. Besides, the frequency, timing, quantity and form of tobacco use among donors vary, making it challenging to assess the effects of blood donors’ smoking on blood components and transfusion recipient outcomes. Therefore, blood components donated by smokers should not be rejected but be subject to additional monitoring [21]. The following three-step strategy may help improve the blood quality and transfusion safety. The first step would be to record the smoking behaviors of blood donors through questionnaires. Secondly, measure cotinine level and COHb content routinely through spectroscopy-based technologies [40, 41]. Thirdly, attach conspicuous labeling of blood components from smokers, avoiding their use in MTP and susceptible recipients, especially pediatric ones [53].

## Conclusion

Smoking has adverse effects on people’s blood circulation. Given the worldwide prevalence of smoking and its potential hazards to blood recipients, improving the management of smokers is one of the pressing tasks to guarantee transfusion safety. Since smoking abstinence among donors is impractical, enhance the monitoring of smokers’ donations seems implementable.

## Data Availability

Not applicable.

## References

[1] Aldosari KH, Ahmad G, Al-Ghamdi S, Alsharif MHK, Elamin AY, Musthafa M (2020). The influence and impact of smoking on red blood cell morphology and buccal microflora: a case-control study. J Clin Lab Anal.

[2] World Health Organization (WHO). Protecting public health requires new tobacco product regulation. 2001. https://www.who.int/news/item/15-05-2001-protecting-public-health-requires-new-tobacco-product-regulation-says-who. Accessed 27 Mar 2020.

[3] Talhout R, Schulz T, Florek E, van Benthem J, Wester P, Opperhuizen A (2011). Hazardous compounds in tobacco smoke. Int J Environ Res Public Health.

[4] Milnerowicz H, Sciskalska M, Dul M (2015). Pro-inflammatory effects of metals in persons and animals exposed to tobacco smoke. J Trace Elem Med Biol.

[5] Martonen TB, Musante CJ (2000). Importance of cloud motion on cigarette smoke deposition in lung airways. Inhal Toxicol.

[6] Berg CJ, Barr DB, Stratton E, Escoffery C, Kegler M (2014). Attitudes toward E-cigarettes, reasons for initiating E-cigarette use, and changes in smoking behavior after initiation: a pilot longitudinal study of regular cigarette smokers. Open J Prev Med.

[7] Munzel T, Hahad O, Kuntic M, Keaney JF, Deanfield JE, Daiber A (2020). Effects of tobacco cigarettes, e-cigarettes, and waterpipe smoking on endothelial function and clinical outcomes. Eur Heart J.

[8] Center for Tobacco Products USFDA. WARNING LETTER—JUUL Labs, Inc. https://www.fda.gov/inspections-compliance-enforcement-and-criminal-investigations/warning-letters/juul-labs-inc-590950-09092019. Accessed 17 Aug 2021.

[9] Grana R, Benowitz N, Glantz SA (2014). E-cigarettes: a scientific review. Circulation.

[10] Jamal A, Phillips E, Gentzke AS, Homa DM, Babb SD, King BA (2018). Current cigarette smoking among adults - United States, 2016. MMWR Morb Mortal Wkly Rep.

[11] General TUSS. Report on e-cigarette use among youth and young adults. Accessed 12 Oct 2021.

[12] Qasim H, Karim ZA, Rivera JO, Khasawneh FT, Alshbool FZ (2017). Impact of electronic cigarettes on the cardiovascular system. J Am Heart Assoc.

[13] Sleiman M, Logue JM, Montesinos VN, Russell ML, Litter MI, Gundel LA (2016). Emissions from electronic cigarettes: key parameters affecting the release of harmful chemicals. Environ Sci Technol.

[14] Cullen KA, Liu ST, Bernat JK, Slavit WI, Tynan MA, King BA (2019). Flavored tobacco product use among middle and high school students - United States, 2014–2018. MMWR Morb Mortal Wkly Rep.

[15] Eaton DL, Kwan LY, Stratton K, editors. Public health consequences of E-cigarettes. Washington (DC), 2018.

[16] Wiencek JR, Gehrie EA, Keiser AM, Szklarski PC, Johnson-Davis KL, Booth GS (2019). Detection of nicotine and nicotine metabolites in units of banked blood. Am J Clin Pathol.

[17] Carson JL, Triulzi DJ, Ness PM (2017). Indications for and adverse effects of red-cell transfusion. N Engl J Med.

[18] Bolton-Maggs PH, Cohen H (2013). Serious hazards of transfusion (SHOT) haemovigilance and progress is improving transfusion safety. Br J Haematol.

[19] Association for the Advancement of Blood and Blood Products (AABB). Standards for blood banks and transfusion services (33rd ed). 2022.

[20] Food and Drug Administration (FDA). Implementation of acceptable full-length and abbreviated donor history questionnaires and accompanying materials for use in screening donors of blood and blood components. https://www.fda.gov/regulatory-information/search-fda-guidance-documents/implementation-acceptable-full-length-and-abbreviated-donor-history-questionnaires-and-accompanying. Accessed 11 May 2023.

[21] Boehm RE, Arbo BD, Leal D, Hansen AW, Pulcinelli RR, Thiesen FV (2018). Smoking fewer than 20 cigarettes per day and remaining abstinent for more than 12 hours reduces carboxyhemoglobin levels in packed red blood cells for transfusion. PLoS One.

[22] World Health Organization (WHO). Report on the global tobacco epidemic. 2021. https://www.who.int/teams/health-promotion/tobacco-control/global-tobacco-report-2021&publication=9789240032095. Accessed 27 July 2021.

[23] Raturi MDV, Kediya A, Chandra S, Kusum A (2021). Advocating optimal abstinence period after smoking although before donating blood. Glob J Transfus Med.

[24] Pretorius E, du Plooy JN, Soma P, Keyser I, Buys AV (2013). Smoking and fluidity of erythrocyte membranes: a high resolution scanning electron and atomic force microscopy investigation. Nitric Oxide.

[25] Asgary S, Naderi G, Ghannady A (2005). Effects of cigarette smoke, nicotine and cotinine on red blood cell hemolysis and their -SH capacity. Exp Clin Cardiol.

[26] Masilamani V, AlZahrani K, Devanesan S, AlQahtani H, AlSalhi MS (2016). Smoking induced hemolysis: spectral and microscopic investigations. Sci Rep.

[27] Hale JP, Winlove CP, Petrov PG (2011). Effect of hydroperoxides on red blood cell membrane mechanical properties. Biophys J.

[28] Norton JM, Rand PW (1981). Decreased deformability of erythrocytes from smokers. Blood.

[29] Gangopadhyay S, Vijayan VK, Bansal SK (2012). Lipids of erythrocyte membranes of COPD patients: a quantitative and qualitative study. COPD.

[30] Pretorius E (2014). Transfusion medicine illustrated. Smokers as blood donors: what do their red blood cell membranes tell us?. Transfusion.

[31] Codandabany U (2000). Erythrocyte lipid peroxidation and antioxidants in cigarette smokers. Cell Biochem Funct.

[32] Leifert JA (2008). Anaemia and cigarette smoking. Int J Lab Hematol.

[33] Boehm RE, Do Nascimento SN, Cohen CR, Bandiera S, Pulcinelli RR, Balsan AM (2020). Cigarette smoking and antioxidant defences in packed red blood cells prior to storage. Blood Transfus.

[34] Attanzio A, Frazzitta A, Vasto S, Tesoriere L, Pintaudi AM, Livrea MA (2019). Increased eryptosis in smokers is associated with the antioxidant status and C-reactive protein levels. Toxicology.

[35] Tzounakas VL, Georgatzakou HT, Kriebardis AG, Papageorgiou EG, Stamoulis KE, Foudoulaki-Paparizos LE (2015). Uric acid variation among regular blood donors is indicative of red blood cell susceptibility to storage lesion markers: a new hypothesis tested. Transfusion.

[36] Yoshida T, Prudent M, D'Alessandro A (2019). Red blood cell storage lesion: causes and potential clinical consequences. Blood Transfus.

[37] Bardyn M, Maye S, Lesch A, Delobel J, Tissot JD, Cortes-Salazar F (2017). The antioxidant capacity of erythrocyte concentrates is increased during the first week of storage and correlated with the uric acid level. Vox Sang.

[38] Marti-Carvajal AJ, Simancas-Racines D, Pena-Gonzalez BS (2015). Prolonged storage of packed red blood cells for blood transfusion. Cochrane Database Syst Rev..

[39] Benowitz NL, Hukkanen J, Jacob P (2009). Nicotine chemistry, metabolism, kinetics and biomarkers. Handb Exp Pharmacol.

[40] DeSimone RA, Hayden JA, Mazur CA, Vasovic LV, Sachais BS, Zhao Z (2019). Red blood cells donated by smokers: a pilot investigation of recipient transfusion outcomes. Transfusion.

[41] DeSimone RA, Plimier C, Lee C, Kanias T, Cushing MM, Sachais BS (2020). Additive effects of blood donor smoking and gamma irradiation on outcome measures of red blood cell transfusion. Transfusion.

[42] Longo LD (1977). The biological effects of carbon monoxide on the pregnant woman, fetus, and newborn infant. Am J Obstet Gynecol.

[43] Aberg AM, Sojka BN, Winso O, Abrahamsson P, Johansson G, Larsson JE (2009). Carbon monoxide concentration in donated blood: relation to cigarette smoking and other sources. Transfusion.

[44] Uchida I, Tashiro C, Koo YH, Mashimo T, Yoshiya I (1990). Carboxyhemoglobin and methemoglobin levels in banked blood. J Clin Anesth.

[45] Ehlers M, McCloskey D, Devejian NS (2003). Alarming levels of carboxyhemoglobin in a unit of banked blood. Anesth Analg.

[46] Ehlers M, Labaze G, Hanakova M, McCloskey D, Wilner G (2009). Alarming levels of carboxyhemoglobin in banked blood. J Cardiothorac Vasc Anesth.

[47] Collard KJ (2014). Transfusion related morbidity in premature babies: possible mechanisms and implications for practice. World J Clin Pediatr.

[48] Valieva OA, Strandjord TP, Mayock DE, Juul SE (2009). Effects of transfusions in extremely low birth weight infants: a retrospective study. J Pediatr.

[49] Davis GL, Gantner GE (1974). Carboxyhemoglobin in volunteer blood donors. JAMA.

[50] Kandall SR, Landaw SA, Thaler MM (1973). Carboxyhemoglobin exchange between donors and recipients of blood transfusions. Pediatrics.

[51] Galazyn-Sidorczuk M, Brzoska MM, Moniuszko-Jakoniuk J (2008). Estimation of Polish cigarettes contamination with cadmium and lead, and exposure to these metals via smoking. Environ Monit Assess.

[52] Nakhaee S, Amirabadizadeh A, Ataei M, Ataei H, Zardast M, Shariatmadari MR (2021). Comparison of serum concentrations of essential and toxic elements between cigarette smokers and non-smokers. Environ Sci Pollut Res Int.

[53] Boehm R, Cohen C, Pulcinelli R, Caletti G, Balsan A, Nascimento S (2019). Toxic elements in packed red blood cells from smoker donors: a risk for paediatric transfusion?. Vox Sang.

[54] Hartwig A (2013). Cadmium and cancer. Met Ions Life Sci.

[55] World Health Organization (WHO). Cadmium Review. 2003. https://www.who.int/publications/m/item/cadmium-review. Accessed 12 Oct 2021.

[56] International Agency for Research on Cancer (IARC). Monographs on the evaluation of carcinogenic risks to humans. 2009. https://monographs.iarc.fr/iarc-monographs-on-the-evaluation-of-carcinogenic-risks-to-humans-19/. Accessed 2 Oct 2021.

[57] Agency for Toxic Substances and Disease Registry (ATSDR). Toxicological profile for lead. 2020. https://www.atsdr.cdc.gov/ToxProfiles/tp13.pdf. Accessed 2 Oct 2021. 36888732

[58] Alvarez-Ortega N, Caballero-Gallardo K, Olivero-Verbel J (2017). Low blood lead levels impair intellectual and hematological function in children from Cartagena, Caribbean coast of Colombia. J Trace Elem Med Biol.

[59] Nascimento SND, Charao MF, Moro AM, Roehrs M, Paniz C, Baierle M (2014). Evaluation of toxic metals and essential elements in children with learning disabilities from a rural area of southern Brazil. Int J Environ Res Public Health.

[60] Lanphear BP, Hornung R, Khoury J, Yolton K, Baghurst P, Bellinger DC (2005). Low-level environmental lead exposure and children's intellectual function: an international pooled analysis. Environ Health Perspect.

[61] Betts KS (2012). CDC updates guidelines for children's lead exposure. Environ Health Perspect.

[62] Caruso RV, O’Connor RJ, Stephens WE, Cummings KM, Fong GT (2013). Toxic metal concentrations in cigarettes obtained from U.S. smokers in 2009: results from the International Tobacco Control (ITC) United States survey cohort. Int J Environ Res Public Health.

[63] Richter P, Faroon O, Pappas RS (2017). Cadmium and cadmium/zinc ratios and tobacco-related morbidities. Int J Environ Res Public Health.

[64] Delage G, Gingras S, Rhainds M (2015). A population-based study on blood lead levels in blood donors. Transfusion.

[65] Gehrie E, Keiser A, Dawling S, Travis J, Strathmann FG, Booth GS (2013). Primary prevention of pediatric lead exposure requires new approaches to transfusion screening. J Pediatr.

[66] AABB Standards Program Committee. Technical manual (19th ed). Bethesda (MD): AABB Press; 2018.

[67] Fusegawa Y, Handa S (2000). Platelet aggregation induced by ADP or epinephrine is enhanced in habitual smokers. Thromb Res.

[68] Fusegawa Y, Goto S, Handa S, Kawada T, Ando Y (1999). Platelet spontaneous aggregation in platelet-rich plasma is increased in habitual smokers. Thromb Res.

[69] Roethig HJ, Koval T, Muhammad-Kah R, Jin Y, Mendes P, Unverdorben M (2010). Short term effects of reduced exposure to cigarette smoke on white blood cells, platelets and red blood cells in adult cigarette smokers. Regul Toxicol Pharmacol.

[70] Van Tiel E, Peeters PH, Smit HA, Nagelkerke NJ, Van Loon AJ, Grobbee DE (2002). Quitting smoking may restore hematological characteristics within five years. Ann Epidemiol.

[71] Padmavathi P, Reddy VD, Maturu P, Varadacharyulu N (2010). Smoking-induced alterations in platelet membrane fluidity and Na(+)/K(+)-ATPase activity in chronic cigarette smokers. J Atheroscler Thromb.

[72] Davis JW, Davis RF (1981). Prevention of cigarette smoking-induced platelet aggregate formation by aspirin. Arch Intern Med.

[73] Davis JW, Hartman CR, Lewis HD, Shelton L, Eigenberg DA, Hassanein KM (1985). Cigarette smoking–induced enhancement of platelet function: lack of prevention by aspirin in men with coronary artery disease. J Lab Clin Med.

[74] Hung J, Lam JY, Lacoste L, Letchacovski G (1995). Cigarette smoking acutely increases platelet thrombus formation in patients with coronary artery disease taking aspirin. Circulation.

[75] Penn A, Keller K, Snyder C, Nadas A, Chen LC (1996). The tar fraction of cigarette smoke does not promote arteriosclerotic plaque development. Environ Health Perspect.

[76] Pretorius E (2012). Ultrastructural changes in platelet membranes due to cigarette smoking. Ultrastruct Pathol.

[77] Opper C, Clement C, Schwarz H, Krappe J, Steinmetz A, Schneider J (1995). Increased number of high sensitive platelets in hypercholesterolemia, cardiovascular diseases, and after incubation with cholesterol. Atherosclerosis.

[78] FitzGerald GA, Oates JA, Nowak J (1988). Cigarette smoking and hemostatic function. Am Heart J.

[79] Takajo Y, Ikeda H, Haramaki N, Murohara T, Imaizumi T (2001). Augmented oxidative stress of platelets in chronic smokers. Mechanisms of impaired platelet-derived nitric oxide bioactivity and augmented platelet aggregability. J Am Coll Cardiol..

[80] Hioki H, Aoki N, Kawano K, Homori M, Hasumura Y, Yasumura T (2001). Acute effects of cigarette smoking on platelet-dependent thrombin generation. Eur Heart J.

[81] Nair S, Kulkarni S, Camoens HM, Ghosh K, Mohanty D (2001). Changes in platelet glycoprotein receptors after smoking–a flow cytometric study. Platelets.

[82] Rubenstein D, Jesty J, Bluestein D (2004). Differences between mainstream and sidestream cigarette smoke extracts and nicotine in the activation of platelets under static and flow conditions. Circulation.

[83] Barua RS, Sy F, Srikanth S, Huang G, Javed U, Buhari C (2010). Effects of cigarette smoke exposure on clot dynamics and fibrin structure: an ex vivo investigation. Arterioscler Thromb Vasc Biol.

[84] Scheinichen D, Heuft HG, Renken C, Juttner B, Jaeger K, Schurholz T (2004). Impact of tobacco smoking on platelet function in apheresis products in vitro. Vox Sang.

[85] Yilmaz S, Eker I, Elci E, Pekel A, Cetinkaya RA, Unlu A (2019). Evaluating the effect of donor anxiety levels and lifestyle characteristics on the activation of platelet concentrates. Blood Res.

[86] Edgren G, Rostgaard K, Vasan SK, Wikman A, Norda R, Pedersen OB (2015). The new scandinavian donations and transfusions database (SCANDAT2): a blood safety resource with added versatility. Transfusion.

[87] Roubinian NH, Kanias T (2019). Blood donor component-recipient linkages: is there fire where there is smoke?. Transfusion.

[88] World Health Organization (WHO). Blood safety and availability. 2017. https://www.who.int/en/news-room/fact-sheets/detail/blood-safety-and-availability. Accessed 15 Aug 2021.

[89] Symvoulakis EK, Vardavas CI, Fountouli P, Stavroulaki A, Antoniou KM, Duijker G (2010). Time interval from cigarette smoke exposure to blood donation and markers of inflammation: should a smoking cut-off be designated?. Xenobiotica.

[90] Hughes JR (1999). Comorbidity and smoking. Nicotine Tob Res.

[91] Merk K, Mattsson B, Mattsson A, Holm G, Gullbring B, Bjorkholm M (1990). The incidence of cancer among blood donors. Int J Epidemiol.

[92] Edgren G, Reilly M, Hjalgrim H, Tran TN, Rostgaard K, Adami J (2008). Donation frequency, iron loss, and risk of cancer among blood donors. J Natl Cancer Inst.

[93] van den Hurk K, Zalpuri S, Prinsze FJ, Merz EM, de Kort W (2017). Associations of health status with subsequent blood donor behavior-an alternative perspective on the healthy donor effect from donor insight. PLoS One.

[94] Atsma F, Veldhuizen I, Verbeek A, de Kort W, de Vegt F (2011). Healthy donor effect: its magnitude in health research among blood donors. Transfusion.

[95] Mousavi SA, Hermundstad B, Saether PC, Nybruket MJ, Knutsen TR, Llohn AH (2021). Health behavior and lifestyle trends among platelet donors: results from a questionnaire-based survey in Norway. Biomed Res Int.

